# Enhancing Fertility in the Same Cycle: A Case Report on Effects of Hysterosalpingo-Foam Sonography (HyFoSy) for a Couple With Mild Infertility

**DOI:** 10.7759/cureus.56495

**Published:** 2024-03-19

**Authors:** Apostolos C Ziogas, Elias Tsakos, Nikolaos Tsagias, Ioannis Thanasas, Polyxeni-Natalia Liasidi, Emmanouil M Xydias

**Affiliations:** 1 Department of Obstetrics and Gynecology, School of Health Sciences, University of Thessaly, Larissa, GRC; 2 Department of Obstetrics and Gynecology, EmbryoClinic IVF, Thessaloniki, GRC; 3 Department of Obstetrics and Gynecology, General Hospital of Trikala, Trikala, GRC; 4 Department of Gynecology, Agios Dimitrios General Hospital, Thessaloniki, GRC

**Keywords:** spontaneous pregnancy, tubal flushing, ultrasound, tubal patency, tubal infertility, hyfosy

## Abstract

Fertility issues are becoming increasingly prevalent, leading many couples to seek fertility treatment at specialized centers. Infertility is a diverse clinical condition, with multiple potential etiologic factors and variable severity in its manifestation. Regardless of the underlying factors and severity, routine fertility assessment rarely differs between cases, with an essential step being fallopian tube patency assessment. Hysterosalpingo-foam sonography (HyFoSy) is the latest available diagnostic technique to assess this parameter, offering robust results, with reduced intra-procedural pain and equipment requirements, in the convenience of the office setting. However, apart from its diagnostic value, HyFoSy has also demonstrated a therapeutic tubal flushing effect, that may be the decisive factor for couples with mild infertility to spontaneously conceive. In this report, we present the case of a couple with mild infertility, who managed to spontaneously conceive after a HyFoSy examination, and in fact within the same cycle.

## Introduction

Infertility is an increasingly prevalent issue in the modern world, affecting up to one in six couples worldwide [1]. Infertility may be caused by a variety of contributing factors, originating from the female side, the male side, or, most frequently, both. One such factor may be fallopian tube mechanical obstruction and/or dysfunction, leading to tubal factor infertility, which is estimated to constitute a contributing factor in over 30% of cases with clinically apparent infertility [2]. At the same time, the presence of tubal obstruction may be the deciding factor between a first-line, simpler fertility treatment (for example, intrauterine insemination - IUI) and a more advanced one (in vitro fertilization or intracytoplasmic sperm injection - IVF/ICSI), not to mention the need for surgery [3]. Therefore, thorough assessment and accurate diagnosis of tubal occlusion is necessary during initial infertility investigation.

Several different tubal patency assessment tests have been implemented in clinical practice, with the more modern, non-invasive methods being preferred, such as Hystero-Salpingo-Graphy (HSG) and Hysterosalpingo-Contrast Sonography (HyCoSy) [4], with the invasive laparoscopic chromopertubation, the gold standard test, being reserved for inconclusive or diagnostically challenging cases [5]. An even newer diagnostic test Hysterosalpingo-Foam Sonography (HyFoSy) was introduced in 2011 [6] with very promising diagnostic performance over the other sonographic method (HyCoSy) [7], while also conferring some practical advantages over HSG, such as increased patient tolerability, protection from ionizing radiation, reduced cost and wider availability [7,8].

Apart from its diagnostic capabilities, HyFoSy has demonstrated the potential for a therapeutic effect on the fallopian tubes and thus a positive effect on fertility, both based on our experience [9], as well as based on findings from the international literature [10]. This effect, while minor compared to proper assisted reproduction technology (ART), may be just enough for mild cases of infertility, with spontaneous conception reported right after HyFoSy was performed, within the same cycle in a few cases [9]. Therefore, for milder cases of infertility who have not yet received any type of ART, HyFoSy may confer a dual advantage, of either boosting fertility just enough to achieve spontaneous pregnancy, forgoing lengthy, costly, and emotionally taxing ART procedures or, failing that, offering essential diagnostic data that will help formulate an appropriate ART treatment plan.

The present report aims at presenting our experience with a case of successful application of HyFoSy as both a first-line diagnostic and therapeutic modality for women with mild infertility, as well as demonstrating its efficacy for immediate results, even within the same cycle.

## Case presentation

A 28-year-old, gravida 0, para 0 woman presented to our clinic with her partner due to infertility after nine months of active attempts. She mentioned that she had already tried timed intercourse four times within those nine months. Other than the clinically apparent infertility, there were no other relevant findings from her personal history. Her BMI was normal (21.76 kg/m^2^). Regarding her gynecological history, she mentioned no menstrual irregularities, with a regular 28-day cycle and a menses duration of five days. She could not recall any pain either during the menses or during intercourse. She had completed her human papillomavirus (HPV) screening (cytology and extended HPV-DNA test within one year, which were both normal). She has no history of surgery or chronic conditions and does not receive any medication on a regular basis, except for a brief history of anxiety attacks in the past, which were treated by escitalopram oxalate (Cipralex™).

Her partner was a 36-year-old man, with a history of prostatitis in the past, for which he received all the proper medication, and a history of appendectomy. With regard to environmental and lifestyle factors, he reported no smoking or alcohol abuse and denies the use of anabolic steroids or other substances. He mentioned no history of chronic disease or allergies. In a semen analysis performed five months prior, all basic analysis parameters (semen volume, sperm concentration, motility, etc.) were within the normal range. However, sperm culture revealed *Escherichia coli *infection, and DNA fragmentation index (DFI) analysis resulted in 34% fragmentation. Antibiotic treatment was administered and the use of anti-oxidant supplements was strongly advised. On follow-up testing after the couple’s visit to our clinic, the infection had fully subsided and most parameters demonstrated a slight improvement (Table 1).

**Table 1:** Comparison of previous and current semen analysis, sperm culture, and DFI assessment results of the male partner. DFI: DNA fragmentation index, *E. coli*: *Escherichia coli*

Parameter	First semen analysis (five months prior)	Second semen analysis (new)
Semen volume (mL)	6.6	4.7
Sperm concentration (10^6^/mL)	32	41
Progressive motility (%)	40	49
Normal morphology (%)	>4	4
Vitality (%)	Not performed	77%
Sperm culture	Positive (*E. coli*)	Negative
DFI (%)	34%	29.2%

The couple had also performed cystic fibrosis genetic testing, both were tested for the DeltaF508 mutation and the woman had also undergone additional mutation testing (99% coverage of known mutations), with normal results for both partners. Additionally, the couple had undergone karyotyping and hemoglobinopathy screening (hemoglobin electrophoresis and sickle cell testing), which were all normal.

**Figure 1:**
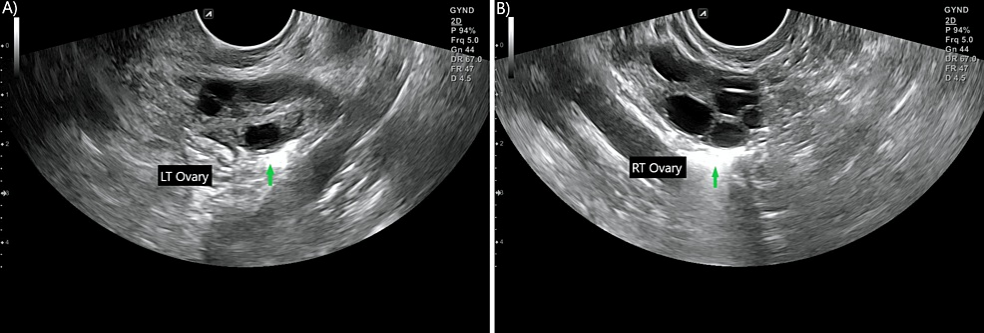
HyFoSy assessment via 2D and 3D (not shown) ultrasonography A: Left tube patent, arrow points at contrast spillage into peritoneum, B: Right tube patent, arrow points at contrast spillage into peritoneum. HyFoSy: Hysterosalpingo-foam sonography

In the context of fertility investigation, a routine gynecological fertility ultrasound was performed. Antral follicle count (AFC) was 25 in total (right ovary: 13, left ovary: 12). The endometrium was of normal appearance without any abnormal findings. Hormonal assessment from a month prior showed follicle-stimulating hormone (FSH): 9.41 mIU/mL, luteinizing hormone (LH): 7.06 mIU/mL, estradiol (E2): 36.20 pg/mL, prolactin (PRL): 18.7 ng/mL, thyroid-stimulating hormone (TSH): 1.24 μIU/mL, and anti-Müllerian hormone (ΑΜΗ): 3.34 ng/mL). Subsequently, HyFoSy was performed to ascertain the patient’s tubal patency. Tubal patency was confirmed bilaterally (Figure 1).

**Figure 2:**
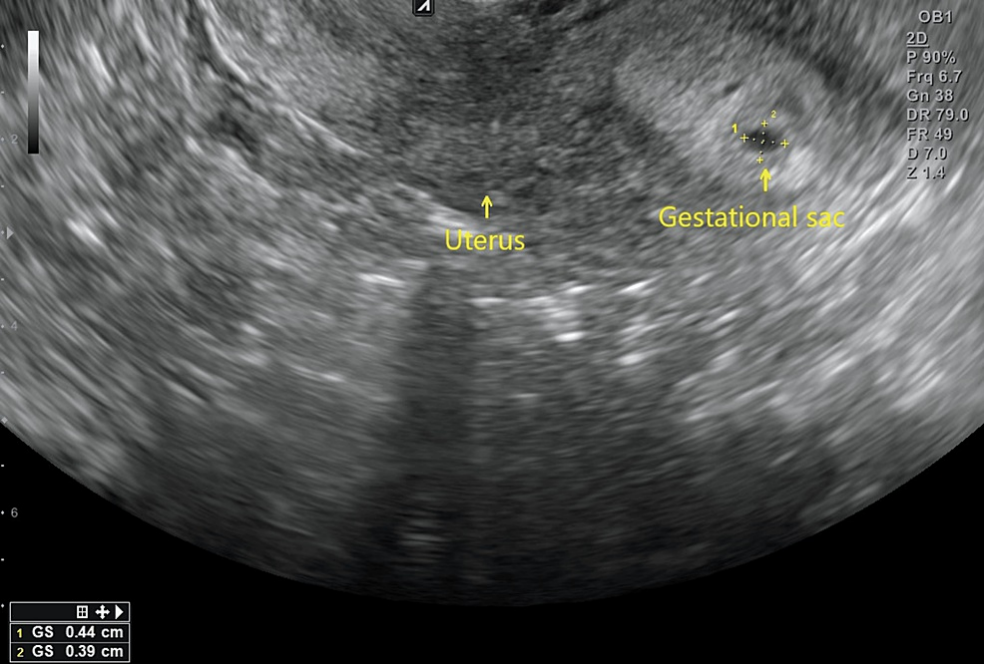
Ultrasonographic confirmation of pregnancy via locating the gestational sac

The couple was then advised to attempt timed intercourse once more and the use of an ovulation test was strongly encouraged. A week after HyFoSy was performed, the woman reported a positive ovulation test, and 18 days after, a positive pregnancy test was reported, with beta human chorionic gonadotropin (beta-hCG) values measured at 907.7 mIU/ml, rising to 2095.7 mIU/ml three days later. On the day of the second beta-hCG measurement, a transvaginal ultrasound scan confirmed the presence of an intrauterine gestational sack (Figure 2). At the time of writing, her pregnancy is progressing uneventfully. A similar therapeutic effect of HyFoSy has been observed previously in our Clinic as well, with another couple with mild infertility experiencing spontaneous conception in the same cycle after HyFoSy examination [9], hinting at a wider trend, worthy of further investigation.

## Discussion

In this study, we presented the case of a young woman and her partner, who, having faced infertility for some time, managed to spontaneously conceive after HyFoSy testing. Furthermore, this was accomplished within the same cycle that HyFoSy was performed, exhibiting the immediacy of this therapeutic intervention.

Tubal flushing and fertility

In general, the fertility-enhancing effect of tubal patency assessment tests on selected patients has been demonstrated before. In fact, in 1966 Wahby et al. [11] were among the first to suggest this fact, demonstrating a 32.2% pregnancy rate after HSG in their patient series. Similar to the present series, they showed that younger age and shorter infertility duration were good prognostic factors for conception after HSG and they reported a mean time of approximately six months to conception, following tubal patency assessment, with same-cycle pregnancy occurring on three occasions [11]. Since then, many more studies have confirmed these findings [12,13].

There have been multiple proposed mechanisms for the fertility-enhancing effect that tubal flushing exerts. The first is the mechanical improvement of patency, via dislodgement of mucinous plugs, cellular debris, or other material that may obstruct the tubal lumen due to the build-up of hydrostatic pressure that ultimately forces the material towards the distal tubal end and into the peritoneal cavity [14-16]. A second proposed mechanism is the exertion of an immunomodulating effect on intraperitoneal leukocytes, as some contrast media have been shown to affect interleukin and prostaglandin production, as well as dendritic and T-regulatory cell function in the endometrium, thus potentially indirectly affecting fertility [14,17]. A similar immunomodulating effect may be occurring within the endometrial cavity as well, via alteration of the cavity’s leukocyte populations and an effect on NK cells, which have been shown to play an important role in implantation and early pregnancy progression [14,18].

Tubal flushing with HyFoSy

Currently, HyFoSy is becoming a more and more popular salpingeal patency assessment test. In contrast with HSG, it involves no exposure to radiation and forgoes the use of iodine contrast media, thus permitting safe use by women with hypothyroidism [19,20]. At the same time, it is reportedly less painful and better tolerated by patients [7,21], less costly, and more widely available, as it can be performed within the Fertility Clinic, using already available ultrasonographic equipment.

As a diagnostic modality, HyFoSy has already demonstrated satisfactory imaging capabilities with regard to fallopian tube patency [8,22]. With regard to its fertility-enhancing effect, HyFoSy showed its therapeutic potential during its introductory study, where the investigators mention that 19% of participants managed to spontaneously conceive, with a median intermittent time of three months [23]. Following that first introduction, many follow-up studies have verified this fertility-enhancing effect. The study by Melcer et al. [24] observed an 11.3% spontaneous pregnancy rate after HyFoSy, with 76% of those occurring within one to three months from the examination date.

Tanaka et al. [25] had a 46.2% pregnancy rate within six months after HyFoSy, with longer duration of infertility being a significant negative predictive factor. Van Schoubroeck et al. [26] assessed 359 women after the examination, with 81 conceiving spontaneously, mostly within the first 1-3 menstrual cycles. Exacoustos et al. [27] calculated a 10.2% spontaneous pregnancy rate within one month after HyFoSy, 29.9% within six months, and 34.4% within one year. A very recent study reported that up to 25% of patients managed to spontaneously conceive, within a year from HyFoSy [28]. While the investigators noted that the mean time between spontaneous pregnancy and HyFoSy was four months, 18.9% of spontaneous pregnancies were noted in the first month [28], similar to the present case. Overall, longer infertility duration (longer than 18 months) and older age were associated with reduced conception rates [25,28].

Our observations are in concordance with the observed trends in the literature, namely the higher spontaneous conception rates within the first one to three months, and higher rates for patients under 35 years old and with infertility duration of less than 18 months [25-28]. In our case, in addition to the aforementioned baseline characteristics, no injective resistance was noticed and patency of both tubes was visualized, both were described as positive predictors for spontaneous pregnancy in the other studies [29].

Our findings on the current case, combined with previous ones [9] and with data from the available medical literature, indicate that there may be specific subgroups of younger patients with mild infertility, who would greatly benefit from the therapeutic effect of HyFoSy, without the need for further escalation to more advanced, costly, physically, and mentally taxing ART procedures. In the future, our team intends to investigate this further, via a larger, prospective cohort study of similar cases to the present report, calculate the rate of and time to spontaneous conception after HyFoSy, evaluate obstetrical outcomes and ultimately ascertain whether HyFoSy could be feasibly offered to this specific patient subgroup as a therapeutic intervention in addition to its diagnostic role.

## Conclusions

In conclusion, HyFoSy is a very promising diagnostic method for tubal patency assessment with many advantages over HyCoSy and HSG. The tubal flushing effect has already been proposed to have a positive impact on fertility patients in the past, with studies demonstrating a similar effect after HyFoSy, an observation confirmed in the present case as well. Research should focus on solidifying the existence and extent of this fertility-enhancing effect, as well as investigating the risk of possible pregnancy or maternal complications after HyFoSy, in order to ensure patient safety and optimal outcomes.
